# Influences of Nanoparticles Characteristics on the Cellular Responses: The Example of Iron Oxide and Macrophages

**DOI:** 10.3390/nano10020266

**Published:** 2020-02-05

**Authors:** Bastien Dalzon, Anaëlle Torres, Solveig Reymond, Benoit Gallet, François Saint-Antonin, Véronique Collin-Faure, Christine Moriscot, Daphna Fenel, Guy Schoehn, Catherine Aude-Garcia, Thierry Rabilloud

**Affiliations:** 1 Grenoble Alpes University, CNRS, CEA, Laboratory of Chemistry and Biology of Metals, BIG-LCBM, 38000 Grenoble, France; Anaelle.torres@cea.fr (A.T.); veronique.collin@cea.fr (V.C.-F.); catherine.aude-garcia@cea.fr (C.A.-G.); 2 Grenoble Alpes University, CNRS, CEA, INAC, SyMMES, RSRM, 38000 Grenoble, France; solveig.reymond@gmail.com; 3 Institut de Biologie Structurale (IBS), University Grenoble Alpes, CEA, CNRS, 38044 Grenoble, France; benoit.gallet@ibs.fr (B.G.); daphna.fenel@ibs.fr (D.F.); guy.schoehn@ibs.fr (G.S.); 4 Grenoble Alpes University, CEA-Grenoble, LITEN, DTNM, L2N, 17 rue des Martyrs, CEDEX 09, 38054 Grenoble, France; francois.saint-antonin@cea.fr; 5 Integrated Structural Biology Grenoble (ISBG) CNRS, CEA, Université Grenoble Alpes, EMBL, 71 Avenue des Martyrs, 38042 Grenoble, France; christine.moriscot@ibs.fr

**Keywords:** macrophage, iron oxide, nanoparticle

## Abstract

Iron oxide nanoparticles/microparticles are widely present in a variety of environments, e.g., as a byproduct of steel and iron degradation, as, for example, in railway brakes (e.g., metro station) or in welding fumes. As all particulate material, these metallic nanoparticles are taken up by macrophages, a cell type playing a key role in the innate immune response, including pathogen removal phagocytosis, secretion of free radical species such as nitric oxide or by controlling inflammation via cytokine release. In this paper, we evaluated how macrophages functions were altered by two iron based particles of different size (100 nm and 20 nm). We showed that at high, but subtoxic concentrations (1 mg/mL, large nanoparticles induced stronger perturbations in macrophages functions such as phagocytic capacity (tested with fluorescent latex microspheres) and the ability to respond to bacterial endotoxin lipopolysaccharide stimulus (LPS) in secreting nitric oxide and pro-cytokines (e.g., Interleukin-6 (IL-6) and Tumor Necrosis Factor (TNF)). These stronger effects may correlate with an observed stronger uptake of iron for the larger nanoparticles.

## 1. Introduction

Determining the toxicity of nanoparticles (NP) is very complex due to the multitude of types of materials, the various mixes of materials, the shape and size polymorphisms [1] and the various coatings which compose NP, etc. However, comparing only the effects that various materials have on cells (e.g., Au-NP vs. Ag-NP or other materials) is not sufficient [2] because the toxicity of particles depends on numerous parameters, such as their aggregation or dissolution, the corona, influenced itself by the particles size [3,4], the shape of particles which influence their kinetic of internalization, their surface charge that may cause bilayer lipidic disorders of cells (positive charge induce high risks of membrane disruption contrary to negative charge) [5], etc. Hence, it is important to go deeper into the field of nanotoxicology and take these parameters into account, but it is almost impossible to evaluate such a variety of parameters in a single series of experiments. In this direction, we sought to assess the possible effects that NP size may have on cell functions for a given nanomaterial, as previously suggested [6]. In this article, we thus assessed the effect of two maghemite/hematite iron oxide nanoparticles (Fe_2_O_3_-NP) of different sizes. Such NPs are commonly found in the urban landscape, for example, in train or metro stations (subway) where particles comprising predominantly Fe_2_O_3_-NP are emitted/released in large amounts due to wheel-rail contact, in particular, during braking [7,8]. Therefore, drivers and railroad workers inhale a large amount of maghemite NP. Train or metro users are impacted, although to a lesser extent. Other occupational exposures to Fe_2_O_3_-NP include welding with the inhalation of welding fumes during the soldering process. These forms of air pollution raise public health questions. As an example, mild steel welding fumes induce inflammation and increase adhesion and infection of bacteria such as Streptococcus pneumonia via the increase of platelet-activating factor (PAFR) in lungs, leading to an increase in pulmonary infections [9].

In order to pursue studies perform on pulmonary cells [10], assessing the effect of NP on macrophages is not meaningless as various nanomaterials are known to be responsible for respiratory illnesses (such as asbestosis or silicosis) and many toxicological studies have found that nanoparticles are able to induce pro-inflammatory responses [11,12,13,14,15] and/or immunological effects [16,17]. In this context, macrophages are of paramount importance in toxicology as (i) they are present in all the tissues of the human body [18,19], (ii) their scavenger function may increase their sensitivity to the effects of NP [20] and (iii) they play a key role in the management of inflammation [21,22]. An impairment of the functionalities of macrophages could cause damage to tissues and induce other immunological diseases (autoimmunity or immune deficiency) [23,24,25,26].

Very different particle sizes can be found in airborne NP present in train stations [27]. Therefore, we decided to investigate the effects of iron oxides nanoparticles of two different sizes (20 and 100 nm) on macrophages at a single and subtoxic exposure concentration of 1 mg/mL. As iron oxide nanoparticles show a strong tendency to aggregate strongly in aqueous solutions, we had to use an organic and biocompatible coating to limit this aggregation. We therefore used a commercially available ferric carboxymaltose nanoparticle (FERINJECT^®^_,_ Vifor Pharma, Bern, Switzerland) for the 20 nm size. FERINJECT^®^ is composed of Fe(III)-oxyhydroxide core obtained through a thermal annealing process described in the original patent [28] and similar to [29] and stabilized/surrounded by a carbohydrate (carboxymaltose) shell which is derived from maltodextrin [30]. We also used a plain 100 nm Fe_2_O_3_-NP purchased from Sigma-Aldrich that we then coated with carboxymaltose. Experiments on the J774A.1 macrophage cell line carried in this manuscript show a significant difference between the way macrophages respond to these two types (and sizes) of iron-based nanoparticles. We show that at the same subtoxic exposure concentration, the larger-sized Fe_2_O_3_-NP produced more effects on macrophages than the smaller-sized ones. In this article, we sought to understand the causes of this difference.

## 2. Material and Methods

Most experiments have been carried out as described in the publications [31,32,33] but details are given in this article to assist in the understanding of this paper. Biological experiments were driven on three independent biological replicates. For cytometry measurement, dead cells were systematically excluded of the assessment with propidium iodide at 1 μg/mL or SytoxRed (Fisher Scientific, Illkirch, France) at 5 nM.

### 2.1. Nanoparticles

Twenty-nanometer ferric carboxymaltose nanoparticles (FERINJECT^®^, 50 mg/mL) were purchased from Vifor Pharma (Bern, Switzerland). One hundred-nanometer maghemite nanoparticles were purchased from Sigma-Aldrich (catalog number: 720704, Sigma-Aldrich, Saint Quentin Fallavier, France), directly as a concentrated suspension at 20% in H2O. We added carboxymaltose in accordance with the patent on FERINJECT^®^ [28] in order (i) to limit aggregation of the particles in the culture medium and (ii) to assess and compare exclusively the effect of size on cells without interferences brought by possible effects of the coating.

### 2.2. Nanoparticle Characterization

The hydrodynamic diameter and particle size distribution were characterized using dynamic light scattering (DLS) after dilution in H_2_O or culture medium DMEM after 0 h or 24 h of incubation at 37 °C, 5% CO_2_. Nanoparticles were diluted at final concentration at 10 µg/mL for measurement. The size and distribution of the particles were assessed after dilution in water or in culture medium by DLS using a Wyatt Dynapro Nanostar machine (Wyatt Technology, Santa Barbara, CA, USA). The morphology of nanoparticles was observed using Transmission Electron Microscopy (TEM) (Thermo Fisher Scientific, Eindhoven, The Netherlands), as previously described [34].

### 2.3. Cell Culture

The mouse macrophage cell line J774A1 was obtained from the European Cell Culture Collection (Salisbury, UK). The cells were cultured in DMEM medium supplemented with 10% fetal bovine serum (FBS). Cells were seeded at 200,000 cells/mL in suspension culture flasks (Greiner Bio-One, Reference 658195, Dutscher, Brumath, France) and harvested at 1,000,000 cells/mL. For treatment with nanoparticles, cells were seeded at 500,000 cells/mL. They were treated with nanoparticles on the following day and analyzed after a further 24 h in culture. In some control experiments, cells were treated with an iron citrate complex (1:2 molarity) prepared for iron (III) sulfate and trisodium citrate, so that an iron concentration in the medium equivalent to the one obtained with 1 mg/mL Fe_2_O_3_ was obtained (i.e., 12.5mM iron). The viability of cells was measured via Facscalibur flow cytometer (BD Biosciences, Le Pont-de-Claix, France) using dye exclusion (propidium iodide at 1 μg/mL or sytoxRed at 5 nM).

Primary macrophages were obtained as described by Dalzon et al. [34].

### 2.4. Particle Internalization Measurement

Qualitative (Perls staining), and quantitative measurement (bathophenantroline and ICP-MS assays) of iron uptake were performed as already described in Dalzon et al. [34].

For TEM- and EDX-microscopy, exposed cells or control cells where fixed for 1 h at room temperature in a fixative solution composed of paraformaldehyde 2% and glutaraldehyde 0.2% in PHEM 0.1 M. Post-fixation was performed during 1 h under shaking in an osmium solution composed of 1% osmium and 1.5% potassium hexaferrocyanate in 0.1 M PHEM buffer. Then, samples were washed with water and stained 30 min under shaking with uranyl acetate 0.5% (in 30% ethanol). Before substitution then impregnation in Embed 812 resin (EPON substitute, EMS), samples were dehydrated in graded series of ethanol (50 to 100%). After polymerisation during 48 h at 65 °C, the blocs were ready to be cut via an Ultramicrotome UC7 (Leica, Rueil-Malmaison, France) in order to produce 80-nm sections. Sections were then collected on formvar-carbon coated copper grids and observed via an FEI Tecnai G2 Spirit BioTwin transmission electron microscope operating at 120 kV with an Orius SC1000B CCD camera (Thermo Fisher Scientific, Eindhoven, The Netherlands) [35,36]. Scanning Transmission Electron Microscopy (STEM) and Energy Dispersive X-rays Spectrum (EDX) (Bruker, Berlin, Germany) for elemental mapping, were achieved via a TECNAI OSIRIS electron microscope (Thermo Fisher Scientific, Eindhoven, The Netherlands) operated at 200 kV and equipped with a 4K GATAN camera (GATAN, Portland, Oregon, USA). The EDX maps were treated with the “ESPRIT” software (version 1.9, Bruker, Berlin, Germany) to minimize the noise.

### 2.5. Phagocytosis Assays

Phagocytosis was assayed by internalization of fluorescent latex beads using flow cytometry as previously described [31,37].

### 2.6. NO and Cytokines Production

In the supernatant culture of cells exposed to NP and activated by LPS, the concentration of NO and cytokines such as interleukin 6 (IL-6), interleukin 10 (IL-10), monocyte chemoattractant protein-1 (MCP-1) and tumor necrosis factor (TNFa) were measured as prevously described [34,38].

### 2.7. F-Actin Staining

Visualization of F-Actin cytoskeleton was assayed by phalloidin staining according to previously published protocols [38,39,40]

### 2.8. Glutathione Assays

Intracellular glutathione levels were assessed using the monochlorobimane technique, with some modifications [33]. Briefly, the cells were harvested, centrifuged during 5 min, and labeled with 75 μM monochlorobimane (diluated in warm PBS) for 5 min at 37 °C. The reaction was stopped via an incubation in ice for 5 min in the dark. The cells were washed twice with cold PBS and finally, the they were analyzed via BD FACSMelodyTM flow cytometer (BD Biosciences, Le Pont-de-Claix, France) using a laser excitation at 405 nm and an emission at 448 ± 45 nm.

### 2.9. Mitochondrial Transmembrane Potential Measurement

The mitochondrial transmembrane potential was assessed using the rhodamine 123 uptake assay, as previously described [40].

### 2.10. Quantitative Bathophenanthroline Assay

The assays were conducted as described in [41] but with some changes. After incubation for 0 h or 24 h at 37 °C, 5% CO_2_ in H_2_O or culture medium, to check the quantity of iron in the two iron-based nanoparticle conditions i.e., FERINJECT^®^ and NP-Fe_2_O_3_ (Sigma, Saint Quentin Fallavier, France), 1 volume of particles (theoretical concentration = 1 mg/mL) or standard solution (Mohr’s salt 17.9 mmol/L) was dissolved with 4 volumes of aqua regia (3:1 volume of 37% hydrocholoric acid and 70% nitric acid). This step required several agitations with vortex mix and a period of incubation of at least 1 h. After dissolution, the samples and standard solution were diluted with water at 1/100. They were then mixed *v*/*v* with a protein precipitant solution (composed of ascorbic acid 2.5 g/L and trichloracetic acid 100 g/L). Then, they were centrifuged at 1500× *g* for 30 min. Finally, an equivalent volume of chromogen solution (composed of bathophenanthroline 0.25 g/L and Na-acetate 123 g/L) was added to the samples. After 10 min of incubation, the absorbance was measured at 535 nm.

For supernatant measurement, 1 mL of iron-based particle suspension was ultracentrifuged at 279,000× *g* for 45 min at 4 °C. The recovered supernatant or standard solution (Mohr’s salt 35.78 µmol/L) was processed as described above from the protein precipitation step to the absorbance measurement step.

For calculating the iron concentration:

$$\frac{\mathrm{Abs}\;\mathrm{sample}\;\mathrm{unknown}\;-\;\mathrm{Abs}\;\mathrm{blank}\;}{\mathrm{Abs}\;\mathrm{iron}\;\mathrm{standard}\;-\;\mathrm{Abs}\;\mathrm{blank}}\;\times \;35.78=\mathrm{concentration}\;\mathrm{of}\;\mathrm{iron}\;\mathrm{µmol}/L\;$$

## 3. Results

### 3.1. Nanoparticle Behavior

**Figure 1:**
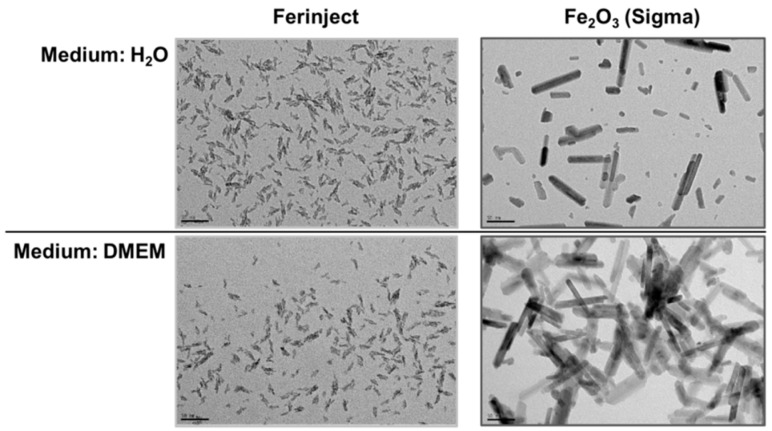
Characteristics of FERINJECT^®^ and Fe_2_O_3_-NP by TEM. Nanoparticles were incubated in H_2_O or DMEM (with carboxymaltose coating for Fe_2_O_3_-NP) after 24 h of incubation at 37 °C, 5% CO_2_. Scale bar 50 nm.

NPs were characterized using DLS and TEM microscopy. Overall, the commercial 20 nm ferric carboxymaltose named FERINJECT^®^ was considered monodisperse (percentage of dispersity < 15%) and had a hydrodynamic diameter comprised between 23 and 25 nm in H_2_O or DMEM. After 24 h of incubation at 37 °C, 5% CO_2_, FERINJECT^®^ was still considered monodisperse and the hydrodynamic diameter is similar to 0 h of incubation (but maybe with a very slightly decreased diameter (1 to 2 nm) in DMEM). The 100 nm Fe_2_O_3_-NP (with carboxymaltose coating) from Sigma-Aldrich had a higher hydrodynamic diameter in H_2_O (both at t0 and after 24 h of incubation) and contrarily to FERINJECT^®^, these NP were strongly agglomerated in the culture medium, with the size of agglomerates > 1 µm of diameter even with the carboxymaltose coating. (Table 1) Examination via Transmission Electron Microscopy (TEM) revealed that FERINJECT^®^ have an irregular shape whereas NP-Sigma are rod-shaped. NP-Sigma have different sizes and we confirmed that they were agglomerated in culture medium (Figure 1).

### 3.2. Fe_2_O_3_-NPs and Iron Uptake by J774A.1

**Figure 2:**
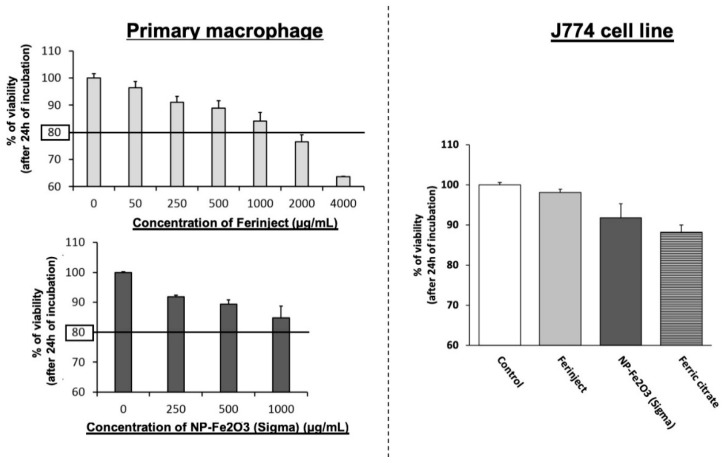
Viability of macrophages. Left graphic: primary mouse macrophages were exposed for 24 h to increasing exposure concentration of FERINJECT^®^ or Fe_2_O_3_-NP Sigma in order to determining LD20. Right graphic: J774A.1 cells lines were incubated with 1 mg/mL of these nanoparticles or equivalent Ferric citrate. Viability was measured using propidium iodide (1 µg/mL).

The effect of the two types of Fe_2_O_3_-NP nanoparticles on cell viability was analyzed and the results are shown in Figure 2. For all the subsequent experiments, a concentration of 1 mg/mL^−1^ was selected, as it is the highest exposure concentration before observing noticeable mortality. We chose this concentration because it corresponds to LD20 (lethal dose 20%) on primary macrophages and allows the testing of functional effects on cells without appreciable cellular mortality. For us, defining an experimental dose similar to environmental exposure is not feasible due to the extreme variability of dose exposure. Taking just the example of a metro station, it depends on the city, the traffic frequency, the age of the metro system and the speed reached, the localization of people: on board or on the subway platform, the type of ventilation, etc. [8,42,43,44] In other words, the chosen a concentration for J774A.1 cells line offered a good compromise between very small cell mortality (J774A.1 were not more affected by Fe_2_O_3_-NP and ferric citrate control, the mortality being below or close to 10%) and the highest probability of observing biological effects.

**Figure 3:**
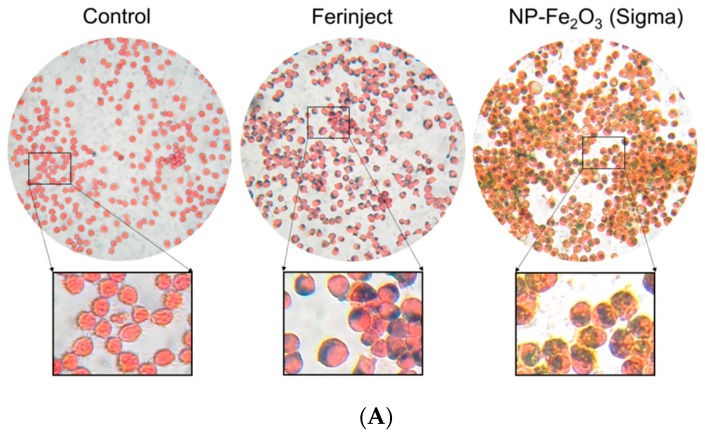

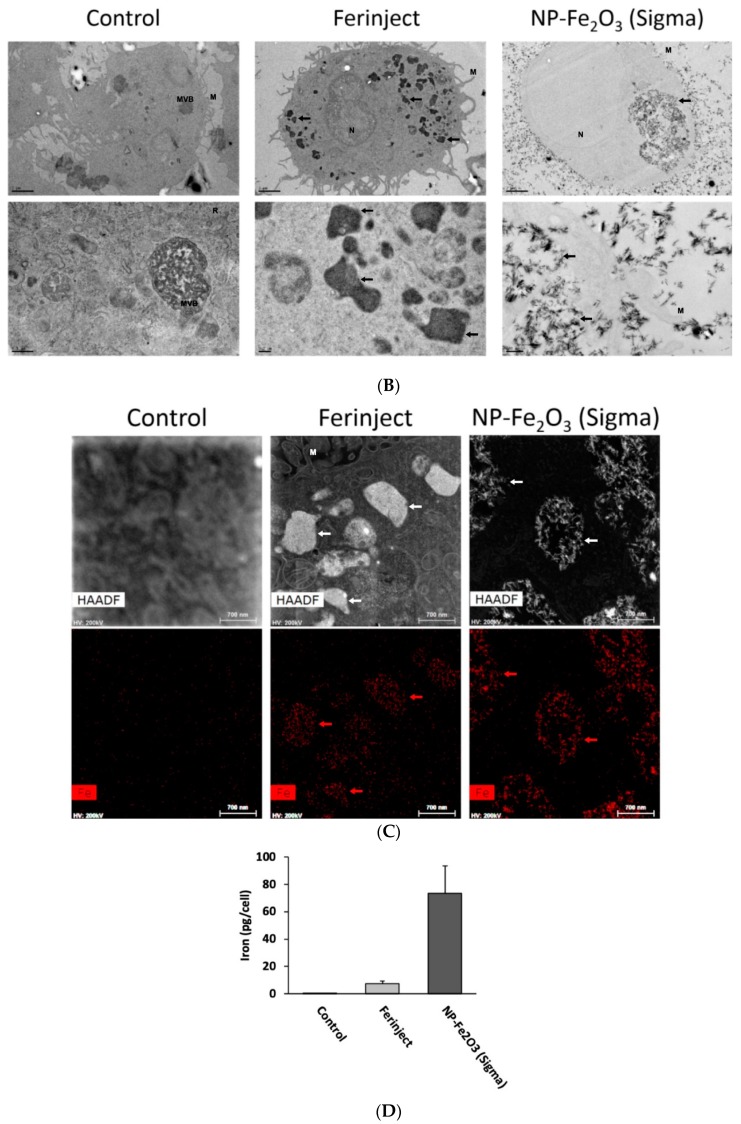
The presence of iron and nanoparticles in J774A.1 cell line incubated 24 h with or without FERINJECT^®^ or Fe_2_O_3_-NP Sigma. Panel (**A**): Perls staining. Blue staining: complex KFe [Fe(CN)6] named Prussian blue. Red staining: Safranin cytosolic staining; Panel (**B**): TEM microscopy. Scale bar, top line = 2 µm; below line = 0.5 µm (Control) or 0.2 µm (FERINJECT^®^ and Fe_2_O_3_-NP Sigma). Panel (**C**): Top line: HAADF (High-Angle Annular Dark Field microscopy) with, in white high-density zone. Below line: EDX (Energy Dispersive X-ray Analysis) with, in red iron elements. N = Nucleus; M = Microvillosity; R = Reticulum; MVB = Multivesicular bodies; arrow = vesicle with iron particles. Panel (**D**): Quantitative assessment of the iron engulfed by macrophages using the bathophenanthroline method.

As a first test, we checked the presence of NPs and iron in cells by microscopy (Perls staining, TEM and EDX microscopy) and a quantitative method (bathophenanthroline assay). Perls staining was used here to reveal the presence of ferric elements inside the cells, as revealed by a prussian blue deposit in J774A.1 cells when cells are incubated with the two types of Fe_2_O_3_-NP (Figure 3A). TEM experiments then showed that NP were present in the vesicles (Figure 3B). TEM also revealed some differences between FERINJECT^®^ and NP-Sigma regarding their uptake. FERINJECT^®^ is present in many vesicles whereas NP-Sigma are always gathered in a single large phagolysosome. EDX microscopy confirmed that iron is associated with both types of Fe_2_O_3_-NP (Figure 3C). Iron loading in the cells was more accurately quantified by the bathophenanthroline assay, which shows that there is an important amount of iron in the cells exposed to Fe_2_O_3_-NP. Our results show a large quantitative difference between FERINJECT^®^ (7.4 pg/cell) and NP-Sigma (73.46 pg/cell) (Figure 3D). The quantification was compared with advanced-technologies such as ICP-MS (Figure S1) which showed results similar to those of the bathophenanthroline assay.

**Figure 4:**
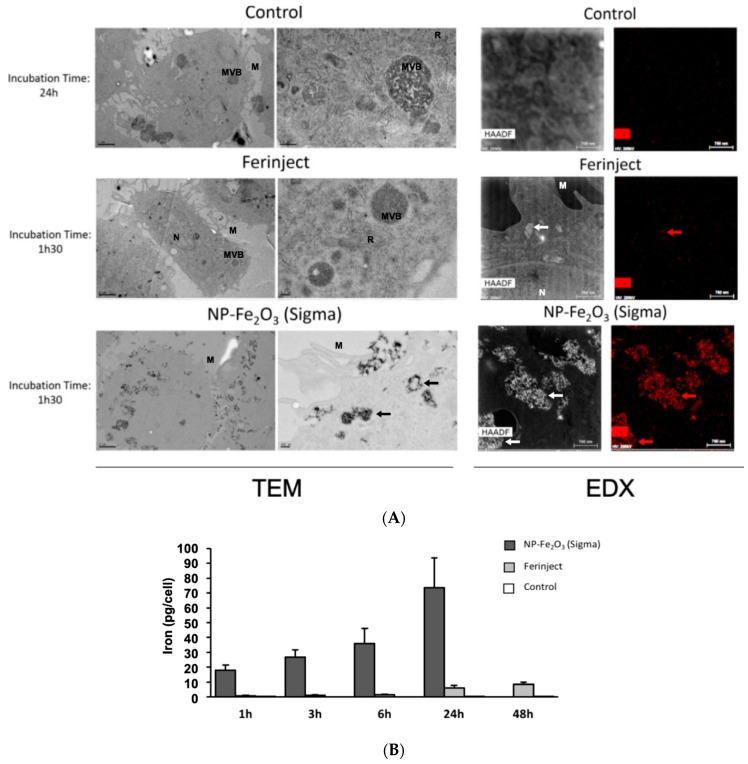
Assessment of Kinetic of iron uptake in J774A.1 cells incubated for various times of incubation with or without FERINJECT^®^ or Fe_2_O_3_-NP. Pannel (**A**): TEM and HAADF-EDX microscopy. J774A.1 were incubated 1h30 with FERINJECT^®^ or Fe_2_O_3_-NP (compared with Figure 3B) TEM scale bar, Left column = 2 µm; Right column = 0.2 µm. HAADF and EDX microscopy scales bar = 0.7µm. N = Nucleus; M = Microvillosity; R = Reticulum; MVB = Multivesicular bodies; arrow = vesicle with iron particles. Pannel (**B**): Quantitative assessment of the Kinetic of iron engulfed by macrophages (1 h to 24 h of incubation with FERINJECT^®^ or Fe_2_O_3_-NP) using the bathophenanthroline method.

We then studied the ability of macrophages to engulf FERINJECT^®^ and NP-Sigma on a kinetic basis. TEM microscopy and EDX microscopy revealed that contrarily to FERINJECT^®^, NP-Sigma are more rapidly internalized by J774A.1 macrophages after a short period of exposure to NP such as 1 h 30 (Figure 4A). In the case of FERINJECT^®^, we only noticed a small (if any) quantity of FERINJECT^®^ in cells while a large quantity of NP-Sigma was already present in the vesicles. We confirmed these results using the quantitative bathophenanthroline assay. After short incubation periods (1 h, 3 h, 6 h), the uptake of NP-Sigma was much higher than that of FERINJECT^®^. Furthermore, the problem of cell detachment observed after 24 h was not encountered after shorter periods of time so that the quantitative determination of iron was more precise in this case. The bathophenanthroline assay (Figure 4B) confirmed what had been observed using TEM microscopy and revealed that NP-Sigma were internalized much faster than FERINJECT^®^ (e.g., internalization of FERINJECT^®^ after 1 h 00 = 0.83 pg/cell, whereas internalization of NP-Sigma = 17.9 pg/cell).

### 3.3. Functional Studies of J774A.1

Functional studies were set up to determine if macrophages in contact with iron particles retained their main biological functions. Phagocytic ability consists in cleaning, for example, apoptotic cells and microorganisms in order to maintain tissue homeostasis. Macrophages should be able to maintain their phagocytic activity even in the presence of Fe_2_O_3_-NP in order to protect the organism against pathogens. On the contrary, the secretion of inflammatory mediators should not be exacerbated by Fe_2_O_3_-NP to avoid damaging healthy tissues (e.g., inflammatory disease). This is why we tested the impact of iron oxide NP on the classical functions of macrophages such as the phagocytic ability, and the modulation of the LPS-induced production of cytokines and NO.

#### 3.3.1. Phagocytic Activity

**Figure 5:**
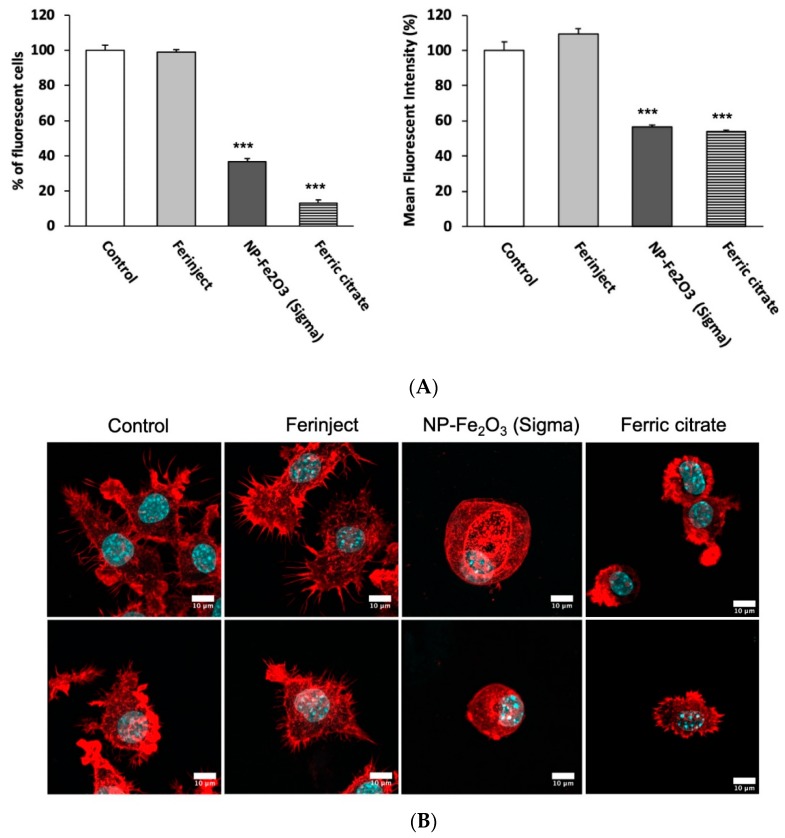
Pannel (**A**): Phagocytic ability. Left graphic: percentage of cells able to phagocyte fluorescent FITC-labeled latex beads (positive cells). Right graphic: phagocytic ability of positive cells. Pannel (**B**): Confocal microscopy (Z-stacks combined): Observation of actin filaments with phalloidin labeled in red (Atto 560). The cell nucleus is colored blue by Dapi. Upper section = apical microscopy view; middle section = center of cell; lower section = basal microscopy view. Statistical confidence (student *t*-test) is indicated as follows *** *p* ≤ 0.001.

Regarding phagocytosis, flow cytometry allows the investigation of two parameters, i.e., the proportion of cells that remain phagocytic after being exposed to NP and the intensity of the phagocytic activity for phagocytosis-positive cells. The results displayed in Figure 5A show that when J774A.1 macrophages were incubated with FERINJECT^®^ for 24 h, their phagocytic capacity was not altered. Contrarily to FERINJECT^®^, NP-Sigma and ferric citrate significantly altered the functionality of J774A.1 macrophages because the phagocytic capacity dropped drastically. Only 37% of cells (NP-Sigma) and 13% (ferric citrate) were able to phagocytize fluorescent beads; furthermore, their phagocytosis ability dropped by 40% to 45% in comparison with the control without NP.

#### 3.3.2. Actin Cytoskeleton

In order to complement the phagocytosis results, we visualized the integrity of the conformation state of the F.actin cytoskeleton (Figure 5B). Indeed, a number of studies using cytochalasin D (which dismantles the actin cytoskeleton) have shown that the conformation of the actin cytoskeleton has a critical role in the phagocytosis process [45,46,47]. When incubated with NP-Sigma, cells were less adherent and showed fewer cytoplasmic elongations than the control without NP (basal view). Moreover, cells were more spherical than the control and showed large vacuoles (middle view). In contrast, we did not observe any differences between the cells incubated with FERINJECT^®^ and control without NP. Ferric citrate cells were generally smaller, less adherent and with fewer cytoplasmic elongations than the control. These results confirm that NP-Sigma and ferric citrate induced damages to the F.actin cytoskeleton, which may explain, at least in part, the results of the phagocytosis assay. Furthermore, in the case of NP-Sigma, we observed a strong vesicularization of the cells that may be linked with an autophagic process [48] (Figure S2).

#### 3.3.3. Secretion of Inflammatory Mediators

**Figure 6:**
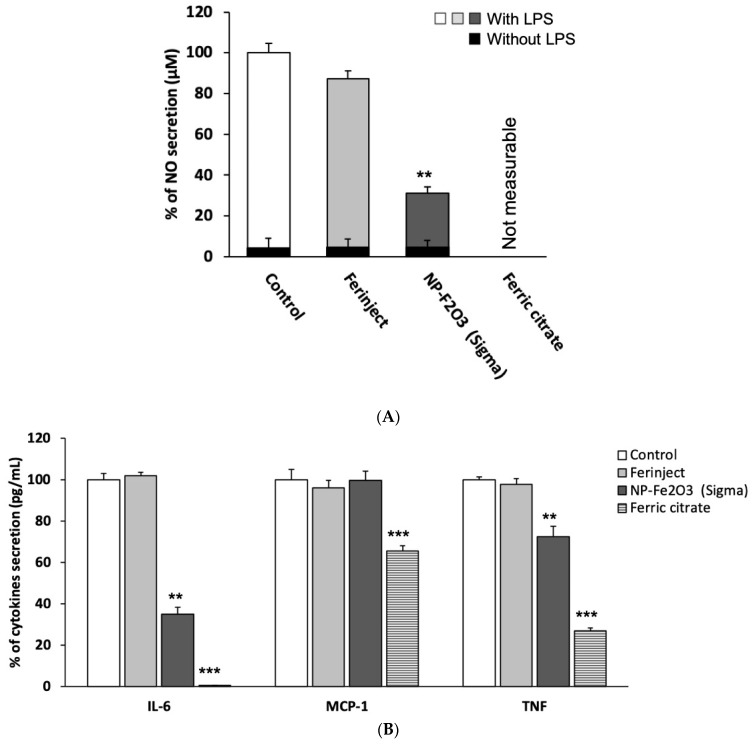
Inflammation ability. Panel (**A**): NO secretion with or without LPS stimulation. Panel (**B**): Secretion of inflammatory cytokines after LPS stimulation. Statistical confidence (student *t*-test) is indicated as follows ** *p* ≤ 0.01; *** *p* ≤ 0.001.

Figure 6A shows that after stimulation with LPS, the secretion of NO was slightly altered in the presence of FERINJECT^®^ as it decreases by only 10%. Conversely, NO secretion drastically decreased (by 69%) for cells exposed to NP-Sigma and was not detectable for cells exposed to ferric citrate because of a strong interference between iron citrate and the Griess reagent. It can be noticed that without stimulation with LPS, NO secretion is significantly lower whatever the NP used. This last result was expected because J774A.1 macrophages were not previously stimulated and therefore, could not mature to inflammatory M1 macrophages. It also showed that iron particles themselves did not induce spontaneous inflammatory signals in J774A.1 cells. A flow cytometric analysis revealed that in the presence of LPS, FERINJECT^®^ did not affect the production of pro-inflammatory cytokines (Figure 6B). The rates of IL-6, MCP-1 and TNF were similar to those of control without NP. However, when cells were incubated with NP-Sigma, the production of pro-inflammatory cytokines was defective for two of the three measured (−65% for IL-6 and −27% for TNF). The secretion of MCP-1 remained unchanged (Figure 6B). The production of all measured cytokines was significantly decreased with ferric citrate.

#### 3.3.4. Cell Physiology Studies: Mitochondrial Potential and Glutathione Level

**Figure 7:**
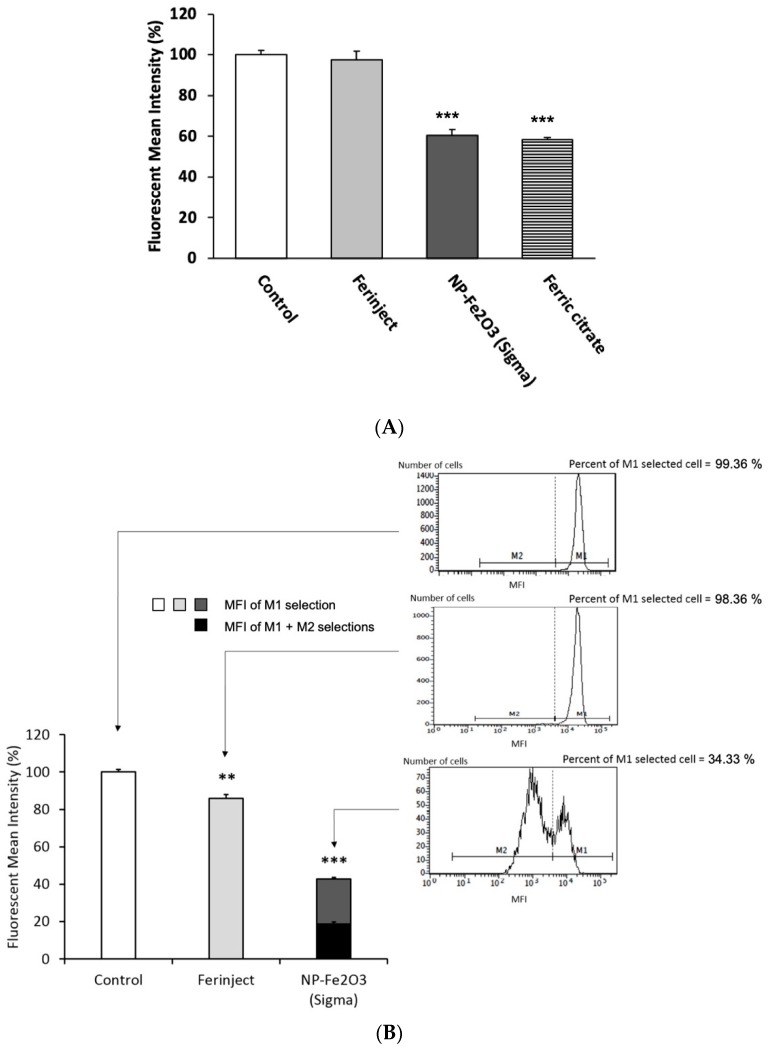
Cell physiology studies of J774A.1 cells incubated 24 h at 37 °C, 5% CO_2_ with or without FERINJECT^®^ or Fe_2_O_3_-NP. Pannel (**A**). Mitochondrial cell assay is measuring fluorescence of Rhodamine 123 accumulated in cells. Analyses with Facscalibur cytometer. Pannel (**B**). Analysis of the glutathione-based antioxidant system. Variations of the intracellular levels of reduced glutathione (GSH). GSH conjugate with monochlorobimane (MCB) form a fluorescent signal which is analysed by Cytometer. Statistical confidence (student’s *t*-test) is indicated as follows ** *p* ≤ 0.01; *** *p* ≤ 0.001.

The various functional studies showed that contrarily to FERINJECT^®^, NP-Sigma had a significant effects on the functionality of macrophages. We tried to obtain further insight into the physiology of macrophages to better understand the differences between the two iron oxide particles. Regarding the mitochondrial membrane potential, our results show that when J774A.1 macrophages were incubated with FERINJECT^®^, their transmembrane mitochondrial potential was not different from that of control, non-exposed cells (Figure 7A), whereas NP-Sigma and ferric citrate caused severe damage to the respiratory ability of J774A.1 macrophages: after exposure to NP-Sigma or ferric citrate, the mitochondrial potential of the cells decreased by 40%. As a consequence, the results suggest that NP-Sigma may induce a decrease in the available energy in the cells. Regarding glutathione, FERINJECT^®^ induced a slight decrease in the rate of free glutathione reduced (GSH) in cells (89% of the control value), whereas with NP-Sigma, it decreased to 43% of the control value (first population) with a second population where the GSH dropped to 19% (Figure 7B). Here again, the test did not work for iron citrate-treated cells, for unknown reasons. The decreased free glutathione content may be due to depletion by metal chelation, as observed for silver [49] and copper [33] nanoparticles. These results suggested that NP-Sigma may alter free glutathione-dependent cellular processes.

## 4. Discussion

Our results point to alterations of the macrophage physiology upon exposure to iron and must first be put into context. Many occupational lung diseases due to the inhalation of particles have been mentioned in the scientific literature (e.g., silicosis and asbestosis caused by crystalline silica or asbestos fibers) [50]. These pathologies enlist macrophages and impair their main functionalities, causing inflammatory and mitochondrial disorders, apoptosis, etc. [25,50,51]. Iron oxide particles are now widespread (e.g., present in metro stations or welding fumes). However, contrarily to silica or asbestos, they can be managed by macrophages [52], but they may be dissolved via acidic phagosomes and may disturb iron homeostasis locally. Indeed, macrophages, in addition to their key role in the initiation and sustainability of the inflammatory response, have a central role in the management of iron homeostasis. This link between inflammatory response, pathogen defense and iron homeostasis control has already been documented [53,54,55,56,57].

In several diseases, due to iron overload, such as hereditary hemochromatosis, an impairment of the main functions of macrophages has been observed, especially in the phagocytic activity (a decrease of 62.5% compared with healthy donors) [58] and in the bactericidal activity via, for example, a deficiency of the secretion of TNF (it is in between 1.9 and 7.0 times lower than the healthy control depending on the period of incubation with LPS) [59]. Concerning pulmonary diseases such as idiopathic pulmonary fibrosis (IPF), an impairment of the functionalities of macrophages, with an alteration of iron homeostasis in pulmonary fibrosis was observed. In this disease, alveolar macrophages are characterized by a deficient transferrin uptake, a reduced phagocytosis of *S. aureus* and an increase of NO production for unstimulated cells [60]. In such diseases, the functional effects observed on macrophages are linked to an overload in soluble iron. In our experiments, we also observed important effect on macrophages functionalities when cells were treated with a high concentration of soluble iron. Interestingly, most of these effects were also observed for macrophages treated with NP-Sigma, but not for cells treated with smaller nanoparticles (FERINJECT^®^). Moreover, these effects were not due to a spontaneous strong dissolution of particles in the culture medium which would release a high quantity of ionic iron. Very little or no iron was measured in the supernatant for all the conditions tested (Table S1). While we did not observe an intrinsic effect of NP-Sigma on NO, as observed in lung fibrosis, we also observed a decrease in phagocytosis for macrophages exposed to these large nanoparticles. Thus, although macrophages are known to manage iron and thus are less sensitive to iron overload and resist to it (low mortality) [61,62], inhaling a high quantity of large iron particles may (i) alter the first line of defense mechanisms of the lungs against pathogens, (ii) promote lung fibrosis, because even if we show that Fe2O3-NP are not toxic at a high concentration (such as 1 mg/mL, LD20 not reached), the functionalities of macrophages can be strongly impacted, and iii) increase bacterial infections which use the excess of iron to develop quicker.

Overall, the uptake kinetics experiments that we performed strongly suggest that the higher effects induced by large particles may be due to the fact that at the end of the 24 h exposure period, cells exposed to larger particles have been exposed to a higher concentration of intracellular iron for a longer time. This observation is in line with sedimentation models to explain the effects of nanoparticles [63]. However, sedimentation is not the only phenomenon that explains the variations in the internalization of particles/nanoparticles. Indeed, in our experiments presented in Figure S3, we assessed the internalization of fluorescent latex beads of 30 nm and 1 µm (corresponding to the size of aggregates of NP-Fe_2_O_3_) and we compared this uptake with phagocytic (J774A.1) and non-phagocytic (MPC11) myeloid cells. This experiment showed that the uptake ratio between J774A.1 and MPC11 changed when we compared the two latex beads sizes. For the 30 nm latex beads, the uptake was similar for J774A.1 and MPC11, while for 1 µm diameter latex beads, the uptake was 20 times more efficient in J774A.1 cells than in MPC11 cells. If the internalization of particle depended solely of the sedimentation phenomenon, the uptake ratios of distinct particles should not be different between the two cell lines. Thus, the final uptake depends not only on sedimentation, which brings the nanoparticles in contact with cells, but also on the internalization ability of the cells, i.e., of the uptake pathways used and their efficiency. In this line, Hslao I-L et al. compared the uptake of 50 nm and 600 nm particles and discussed the differences in their uptake processes. Small particles are preferentially internalized by pinocytosis if not passive diffusion pathways (low energy consumption), while large particle are engulfed by active internalization (high energy consumption) [64].

Nevertheless, although important cell parameters such as the mitochondrial transmembrane potential and the intracellular glutathione content are deeply affected by the large particles, the cells are still alive, and retain some level of functionality. However, the strong effects of large Fe_2_O_3_-NP observed on glutathione and mitochondria resemble ferroptosis, in which the observed effects are linked to the quantity of iron into cells [65].

The present article highlights that contrarily to the received opinion and some articles, nanoparticles are not systematically more noxious (or more toxic) than larger particles such as microparticles. Of course, not all materials react in a similar manner and a lot of parameters influence the toxicity of nanoparticles [66]. Given that the toxicity of NPs depends on a multitude of parameters, the nanotoxicology discipline is extremely complex [67] and the analysis of one peculiar group cannot be generalized to all nanoparticles. Regarding our in vitro study, even if the larger Fe_2_O_3_ microparticles induce more effects than nanoparticles, they are not necessarily more toxic in vivo. In fact, nanoparticles, because of their small size, can enter the respiratory system more deeply (e.g., pulmonary alveoli) [68,69] and seem to cross biological barriers (mucus, surfactant, etc.) more easily [70] and are thus more likely to enter the bloodstream via the respiratory tract. Furthermore, particles with a large size are more easily removed by exhalation [63] whereas small particles are eliminated by phagocytosis via the macrophages or accumulate in the alveolae [71]. Therefore, it is more difficult to eliminate them from the body and they are more likely to persist in the lungs. According to a study by TSI Incorporated, when 20 nm nanoparticles are inhaled, 40% to 60% are found in the alveolae.

It must, however, be mentioned that according to the same study (measuring nanoparticle exposure application note of Thermo-System Inc. via the Nanoparticle Surface Area Monitor (Application Note NSAM-001), 10% to 20% of inhaled large particles (≥100 nm) are deposited in the alveoli. [72]. Thus, even if large particles are less present in alveolar regions, their quantity is not negligible, particularly when a high quantity is released into the air (which is the case of metro stations during braking). Moreover, the previously mentioned figures are numbers of particles. As large particles imply a considerably higher mass of material than smaller ones at equal numbers, it is therefore highly relevant to investigate the toxicity of large particles.

Even if we cannot actually conclude which of the two particles is more toxic in real life due to lack of in vivo data, it remains interesting to note that at a same concentration, two iron based particles with different sizes and aggregate forms do not induce the same effects on macrophages. Moreover we show that even without inducing important cell death, particles can have a drastic impact on the functionalities of macrophages and thus, may facilitate the occurrence of pathologies, as observed for welding fumes [9], e.g., by decreasing the overall efficiency of the immune system. Finally, in real-life conditions, nanoparticles are not isolated but on the contrary, exist alongside a variety of other elements mix (particulate, nanoparticulate or other chemical species). Therefore, in order to build on our results, we plan to pursue with cross-toxicity studies which, like existing experiments on other nanoparticles by [20,31], better reflect real-life conditions. We also aim at investigating the potential cross-toxicity between exposure to nanoparticles and certain aspects of lifestyle (e.g., cigarette smoke, etc.). [73,74]

## Figures and Tables

**Table 1:** Characterization of FERINJECT^®^ and Fe_2_O_3_-NP Sigma by DLS. Nanoparticles were incubated in H_2_O or DMEM (with carboxymaltose coating for Fe_2_O_3_-NP Sigma) after 24 h of incubation at 37 °C, 5% CO_2_.

FERINJECT^®^					Fe_2_O_3_-NP (Sigma)			
Medium	H_2_O		DMEM		H_2_O		DMEM	
Incubation time	0 h	24 h	0 h	24 h	0 h	24 h	0 h	24 h
Size (nm)	23.4	22.4	24.3	21.9	73.4	79.6	1258	1120
Dispersity (%)	8.8	11.7	8.5	12.2	22.4	22.6	Multimodal	Multimodal

## Supplementary Materials

The following are available online at https://www.mdpi.com/2079-4991/10/2/266/s1, Figure S1: Quantitative assessment of the iron, Figure S2: Confocal microscopy and, Figure S3: Internalization of FITC-labeled latex beads by MPC11 or RAW for 24 h at 37 °C, 5% CO2. Figure S4: Internalization of FITC-labeled latex beads by MPC11 or RAW for 24h at 37 °C, 5%CO2. Table S1: Quantitative bathophenanthroline assay.
