# Supplementary material for: Influences of Nanoparticles Characteristics on the Cellular Responses: The Example of Iron Oxide and Macrophages
Source: Nanomaterials (Basel). 2020 Feb 5;10(2):266. doi: 10.3390/nano10020266 (PMC7075185; doi:10.3390/nano10020266)
Supplement: Supplementary file 1 [file nanomaterials-10-00266-s001.pdf]

nanomaterials

MDPI

Supplementary data:

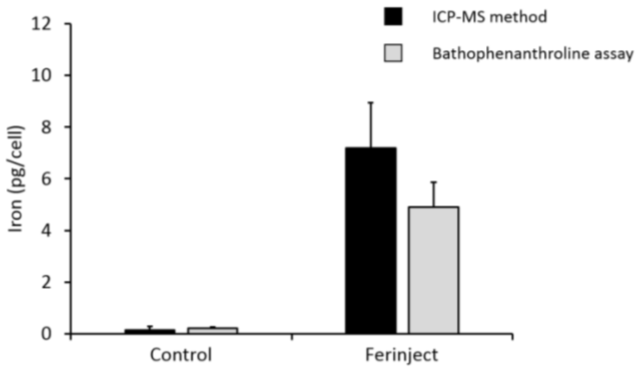

Figure S1. Quantitative assessment of the iron: validation of bathophenanthroline by comparing with another quantitative measurement which is ICP-MS method.

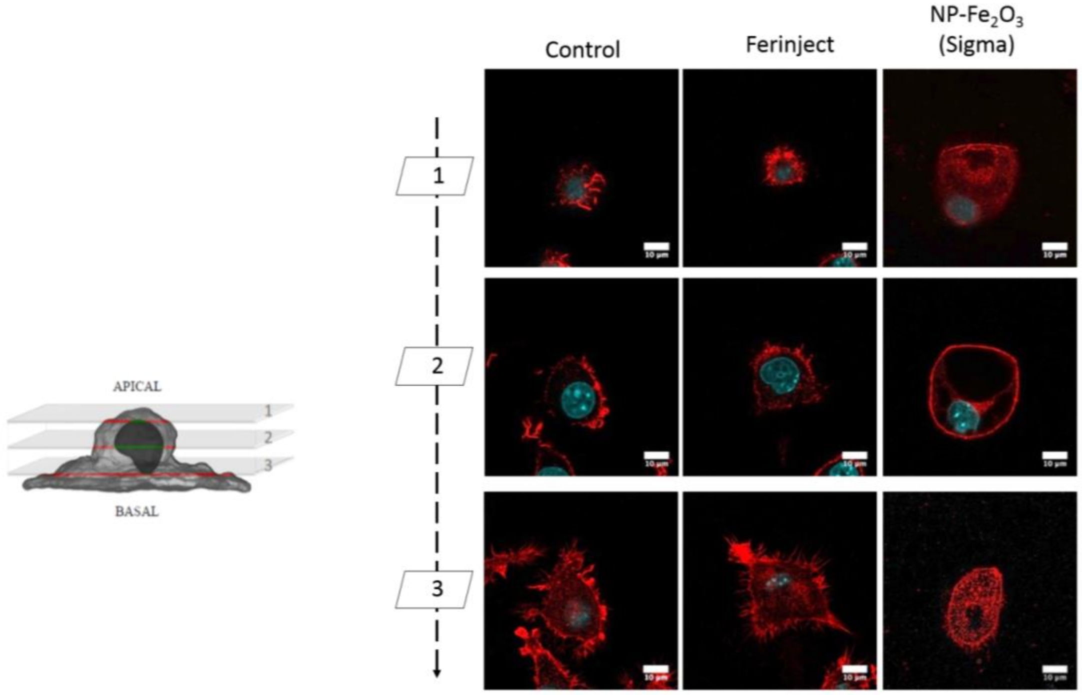

Figure S2. Confocal microscopy. Observation of actin filaments with phalloidin labeled in red (Atto 560). Cell nucleus is colored blue by Dapi. Upper section = apical microscopy view; middle section = center of cell; lower section = basal microscopy view.

Table S1. Quantitative bathophenanthroline assay. Iron based particles (FERINJECT or NP-Fe2O3 (Sigma)) was incubated 0h or 24h at 73°C, 5% CO2 in two different medium (H2O or culture medium + 10% FBS). For all incubation condition, iron in samples (particle + supernatant) or only supernatant were measured. Limit of detection = $0{,}1 \times 10^{-3}$ mg/mL.

Medium : H2O			
	total of sample (Nanoparticle dissolved + supernattant)	Supernattant After 0h of incubation	Supernattant 24h of incubation
Ferinject [iron] mg/mL	1,1 ± 0,37	$0{,}67 \times 10^{-3} \pm 0{,}19 \times 10^{-3}$	$1{,}05 \times 10^{-3} \pm 0{,}4 \times 10^{-4}$
NP-Fe2O3 (Sigma) [iron] mg/mL	1,05 ± 0, 34	$0{,}7 \times 10^{-3} \pm 0{,}19 \times 10^{-3}$	$0{,}97 \times 10^{-3} \pm 0{,}26 \times 10^{-3}$
**Medium : Culture medium**			
	total of sample (Nanoparticle dissolved + supernattant)	Supernattant After 0h of incubation	Supernattant 24h of incubation
Ferinject [iron] mg/mL	1,19 ± 0,17	$1{,}4 \times 10^{-3} \pm 0{,}29 \times 10^{-3}$	$0{,}23 \times 10^{-3} \pm 0{,}19 \times 10^{-3}$
NP-Fe2O3 (Sigma) [iron] mg/mL	0,85 ± 0,017	undetectable	undetectable

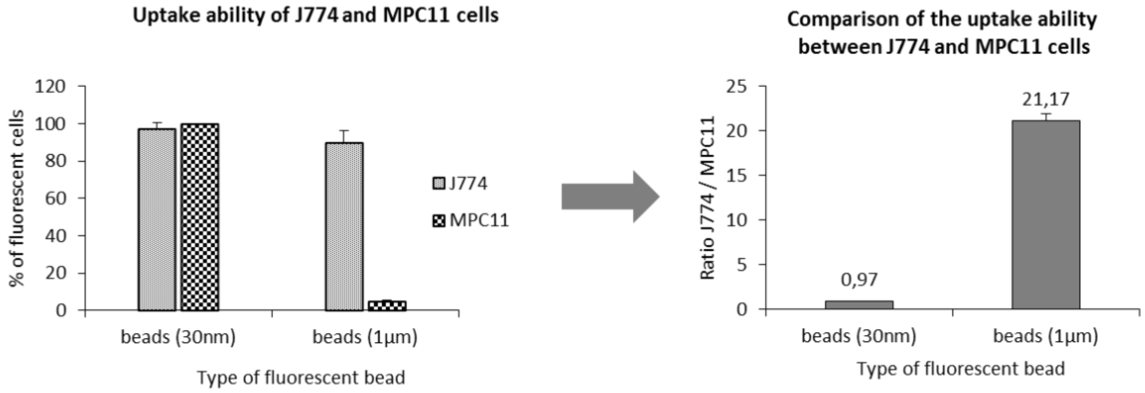

Figure S4. Internalization of FITC-labeled latex beads by MPC11 or RAW for 24h at 37°C, 5%CO2. 30 nm or 1 µm FITC-labeled latex beads were used. Left graphic: Mean fluorescence intensity (MFI) of cells (MPC11 non-phagocytic cells or J774 phagocytic cells) measured by Facscalibur Cytometer. Right Graphic: Ratio of MFIJ774 / MFIMPC11.
